# Considerations for viral co-infection studies in human populations

**DOI:** 10.1128/mbio.00658-24

**Published:** 2024-06-07

**Authors:** Taylor Chin, Ellen F. Foxman, Timothy A. Watkins, Marc Lipsitch

**Affiliations:** 1 Center for Communicable Disease Dynamics, Harvard T.H. Chan School of Public Health, Boston, Massachusetts, USA; 2 Department of Epidemiology, Harvard T.H. Chan School of Public Health, Boston, Massachusetts, USA; 3 Department of Laboratory Medicine, Yale University School of Medicine, New Haven, Connecticut, USA; 4 Department of Immunobiology, Yale University School of Medicine, New Haven, Connecticut, USA; Albert Einstein College of Medicine, Bronx, New York, USA; McMaster University, Hamilton, Ontario, Canada

**Keywords:** epidemiology, virus–virus interactions, selection bias, co-detections

## Abstract

When respiratory viruses co-circulate in a population, individuals may be infected with multiple pathogens and experience possible virus–virus interactions, where concurrent or recent prior infection with one virus affects the infection process of another virus. While experimental studies have provided convincing evidence for within-host mechanisms of virus–virus interactions, evaluating evidence for viral interference or potentiation using population-level data has proven more difficult. Recent studies have quantified the prevalence of co-detections using populations drawn from clinical settings. Here, we focus on selection bias issues associated with this study design. We provide a quantitative account of the conditions under which selection bias arises in these studies, review previous attempts to address this bias, and propose unbiased study designs with sample size estimates needed to ascertain viral interference. We show that selection bias is expected in cross-sectional co-detection prevalence studies conducted in clinical settings, except under a strict set of assumptions regarding the relative probabilities of being included in a study limited to individuals with clinical disease under different viral states. Population-wide studies that collect samples from participants irrespective of their clinical status would meanwhile require large sample sizes to be sufficiently powered to detect viral interference, suggesting that a study’s timing, inclusion criteria, and the expected magnitude of interference are instrumental in determining feasibility.

## OVERVIEW OF VIRUS–VIRUS INTERACTIONS

Seasonal cocirculation of respiratory viruses may result in individuals being infected concurrently or sequentially with multiple pathogens. In this context, virus–virus interaction is often broadly defined as the phenomenon where concurrent or recent prior infection with one virus affects the infection process of another virus (1). Interactions may be classified as positive (synergistic) if infection with one virus promotes the presence of another virus or negative (antagonistic) if infection with one virus impedes the presence of another virus. An additional classification described in the literature distinguishes homologous and heterologous interference within negative virus–virus interactions based on whether two viruses are antigenically distinct (1).

In a null model in which two viruses A and B transmit independently from one another, knowledge that an individual harbors one virus would provide no information about whether they harbor the other—that is, if we characterize viruses A and B by their presence or absence in a population,


(1) $$P\left(A|B\right)=P\left(A|\overset{-}{B}\right)=P\left(A\right)$$

If equation 1 is not true, there could be several explanations for an observed statistical association between viruses A and B: (i) virus A promotes or inhibits virus B; (ii) virus B promotes or inhibits virus A; (iii) there is a common cause, *L*, of infection or non-infection with viruses A and B (also known in epidemiology as a confounder of the causal relationship between the infection processes of viruses A and B); or (iv) the sample has been collected in a way that conditions on a common effect, *C*, of two variables—one of which is virus A or a variable associated with virus A and the other is virus B or a variable associated with virus B (also known as selection bias or collider-stratification bias) (2).

Whereas explanations i and ii reflect interactions within an individual between the infection processes of two viruses, explanations 3iii and iv do not. Studies attempting to examine phenomena i and 2ii should therefore be designed to minimize bias from confounding or selection bias. The causal mechanisms underlying explanations i and ii can be due to “biological/direct” mechanisms (e.g., antiviral role of activated interferon-stimulated genes) (3–9) and/or “behavioral/indirect” mechanisms that lead to people infected with virus A not getting infected with virus B (e.g. they are more likely to stay at home when feeling unwell). Age and immunocompetence are examples of explanation iii or common causes of the risk of infection of each of the two viruses that have been discussed in the literature (10, 11).

With regard to explanation iv, various studies have attempted to ascertain virus–virus interactions at the population level using cross-sectional co-detection prevalence studies, aided by the development of multiplex PCR (3, 12–15). In this study design, the expected proportion of co-detections of two viruses under the null model of independence is calculated as the product of the individual prevalence of the two viruses. That is, if the prevalence of virus A is *P*(A) and the prevalence of virus B is *P*(B) in the population, then under the null hypothesis of no interaction, in the absence of confounding or selection bias, the expected proportion of the population infected with both viruses would be *P*(A)*P*(B) in an unbiased sample of the population. If the observed number of co-detections is significantly less than this expected number, negative interaction, or viral interference, is inferred.

A key to this study design is the assumption that an unbiased sample of the population is used, that is, a sample in which the prevalence of each virus and of the two together is the same as that in the source population. Co-detection studies, however, are often conducted using clinical samples from people with acute respiratory illness (ARI) symptoms due to availability of data from these populations. Selection bias, or collider-stratification bias, is a common methodological issue with this type of study (2). An intuitive way to think about the bias is that the sample of patients presenting to clinical care with ARI symptoms is likely to have higher proportions of individuals with at least one of the respiratory viruses than the population from which it is drawn. It has therefore been correctly argued that finding a departure from independence in such a sample—a reduced proportion of co-infected compared to the expected proportion based on the prevalence of singly infected individuals—may reflect only this biased sampling and not necessarily any underlying antagonism between viruses in the population (16–18). Table 1 summarizes different possible explanations for an observed statistical association between viruses A and B. While it is beyond the scope of this article to discuss all possible explanations, the various explanations summarized in Table 1 motivate the need for carefully designed and analyzed population-level studies that seek to separate out the possible causes for an observed statistical association.

**TABLE 1:** Possible explanations for an observed statistical association between the cross-sectional prevalence of two viruses [i.e., *P*(A, B) ≠ *P*(A)*P*(B), where *P*(A, B) is the prevalence of infection with both viruses simultaneously]

Explanation for statistical association	Diagram of one such explanation	Explanation’s effect on infection with virus B among individuals infected with virus A or vice versa	Relevant literature
Direct interference: virus A infection causes changes in target cells that inhibit infection with virus B and/or vice versa^*a*^	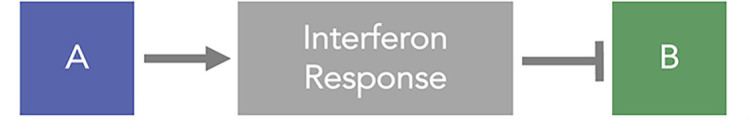	Reduces prevalence of coinfection	(3–5, 10, 19)
Direct potentiation: virus A infection causes changes in target cells that promote infection with virus B and/or vice versa^*a*^	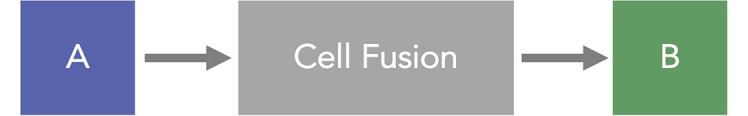	Increases prevalence of coinfection	(10, 20, 21)
Indirect interference: virus A infection reduces contact rates, thereby reducing probability of acquiring virus B, and/or vice versa^*a*^	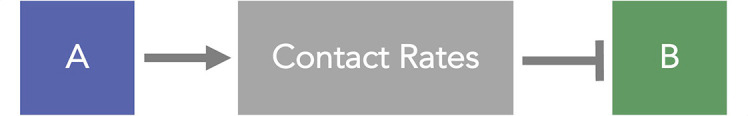	Reduces prevalence of coinfection	(22)
Cross-immunity potentiation: adaptive immunity from past infection with virus A increases susceptibility to virus B and/or vice versa	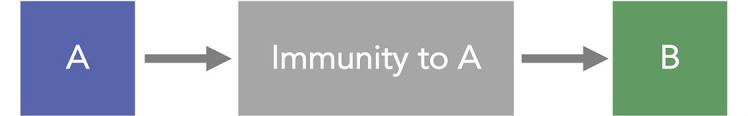	Increases the frequency of viruses A and B at different times in the same individual, thereby decreasing the relative frequency of coinfection	(10, 23)
Cross-immunity interference: adaptive immunity from past infection with virus A decreases susceptibility to virus B and/or vice versa	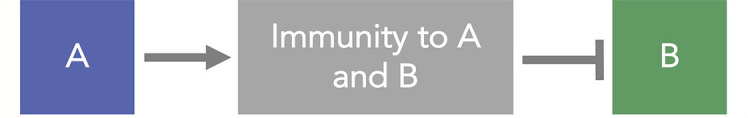	May increase the prevalence of coinfection, although the incidence of coinfection decreases	(10)
Confounding: a common determinant of infection with viruses A and B creates a non-causal association between them. This could include nearly any complexity of host demography/clinical state, space, or time not fully accounted for in statistical models (10).	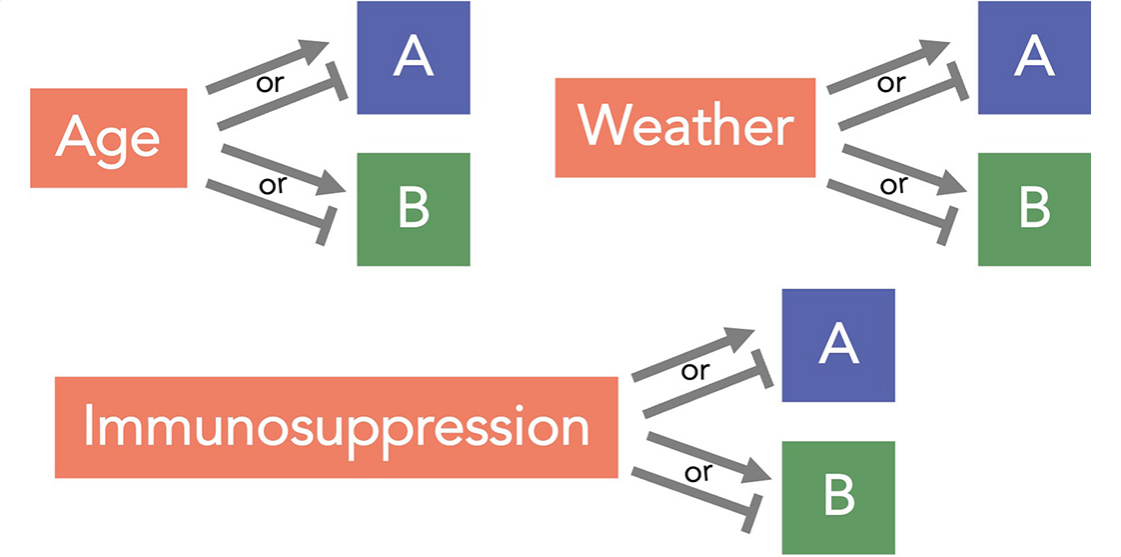	Reduces or increases prevalence of coinfection, depending on the confounder	(10, 11)
Selection bias via a sub-multiplicative effect, which means that compared to someone with neither virus, the relative risk of being selected into the study population when infected with both viruses is less than the product of the relative risks of being selected into the study population from being infected with each virus individually.	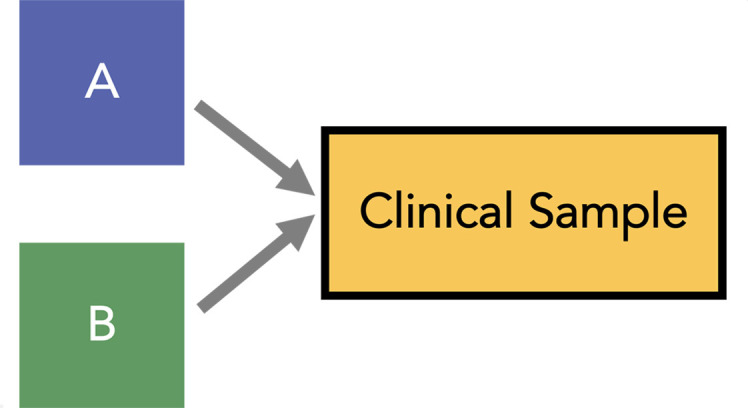 . .	Reduces prevalence of coinfection when only subjects with certain characteristics are studied (e.g., only subjects with ARI symptoms are included)	(16–18)
Selection bias via a supra-multiplicative effect, which means that compared to someone with neither virus, the relative risk of being selected into the study population when infected with both viruses is greater than the product of the relative risks of being selected into the study population from being infected with each virus individually.	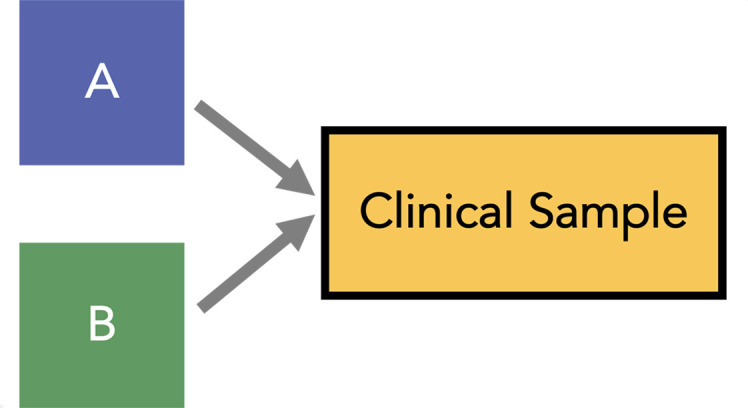	Increases prevalence of coinfection when only subjects with certain characteristics are studied (e.g., only subjects with ARI symptoms are included)	Hypothetical

^*a*^ Note that if the sign of the interaction is opposite for A’s effect on B and B’s effect on A, the overall effect on the probability of co-infection depends on additional factors (10).

The SARS-CoV-2 pandemic has regenerated interest in studying virus–virus interactions because of their implications for disease forecasting and evaluation of interventions. This article will focus specifically on the selection bias issues associated with co-detection prevalence studies in samples drawn from clinical settings. We choose this focus because the simplicity of this study design has made it common in the literature. Here, we first provide a quantitative account of the conditions under which selection bias arises in co-detection studies and its mechanistic interpretation, then discuss previous attempts to address this bias, and, last, propose unbiased study designs with estimates of the sample sizes needed to ascertain viral interference. We focus on virus–virus interactions in the form of interference due to recent studies that have motivated this article, but the same arguments equally apply to designing studies to evaluate potentiation of one viral infection by another.

## DEFINING THE SELECTION BIAS ISSUE IN CO-DETECTION STUDIES

Let the population odds ratio (OR), ${OR}_{X}$*,* be defined as the ratio of the odds of having virus A in the presence of virus B, relative to the odds of having virus A in the absence of virus B, in the population:


(2) $$OR_{X}=\frac{\left[\frac{P(A|B)}{P(\bar{A}|B)}\right]}{\left[\frac{P(A|\bar{B}}{P(\bar{A}|\bar{B})}\right]}=\frac{\left[\frac{P(A,B)}{P(\bar{A},B)}\right]}{\left[\frac{P(A,\bar{B}}{P(\bar{A},\bar{B})}\right]}$$

where $\overset{-}{A\;}$ denotes the absence of virus A and $\overset{-}{B\;}$ denotes the absence of virus B. Note that this is equivalent to the simple OR of the 2 × 2 table for presence/absence of A and B, which is the second expression in equation 2.

If ${OR}_{X}<1$, the odds of having virus A in the presence of virus B are less than the odds of having virus A in the absence of virus B. This is symmetric, in that ${OR}_{X}<1$ also indicates that the odds of having virus B in the presence of virus A are less than the odds of having virus B in the absence of A.

In reality, ${OR}_{X}$ is rarely if ever observed because only a sample of individuals, not the whole population, is assayed for viral infection in any particular study. In practice, that study sample often consists of patients who report ARI symptoms and present to a clinical setting, where they receive molecular testing.

Let ${OR}_{Y}$ be defined as the ratio of the odds of having virus A given they have virus B and are included in the study population, represented by S*,* relative to the odds of having virus A given they do not have virus B and are included in the study population:


(3) $${OR}_{Y}=\left[\frac{P\left(A|B,\;S\right)}{P\left(\overset{-}{A}|B,\;S\right)}\right]/\left[\frac{P\left(A|\overset{-}{B},S\right)}{P\left(\overset{-}{A}|\overset{-}{B},\;S\right)}\right]$$

Using Bayes’ theorem, the population OR, ${OR}_{X},$ can be written as the following:


(4) $${OR}_{X}={OR}_{Y}/M,$$

where


(5) $$M=\left[\frac{P\left(S|A,\;B\right)}{P\left(S|\overset{-}{A},\;B\right)}\right]/\left[\frac{P\left(S|A,\overset{-}{B}\right)}{P\left(S|\overset{-}{A},\;\overset{-}{B}\right)}\right]$$

Conceptually, the probability terms of *M* represent the relative selection probabilities of people being included in the study based on their viral status, and *M* is the cross-product of these selection probabilities.

Greenland (24) referred to *M* as a selection bias factor and pointed out that a corrected estimate, here the unbiased population-level OR, ${OR}_{X}$ , which is the estimand of interest, could be obtained by dividing the biased sample OR, ${OR}_{Y}$ , by the selection bias factor, per equation 4.

Knowledge of *M* would be sufficient to obtain an unbiased estimate of ${OR}_{X}$—the population-level OR, with all other assumptions holding for valid inference previously described. The key obstacle to this approach, however, is determining the selection probabilities in equation 5 in the first place, as they are generally unknown.

Looking at equation 4*,* we can understand somewhat intuitively the circumstances under which selection bias can arise when conducting a study of patients presenting for health care for ARI. (For shorthand, we use “ARI symptoms” in the rest of the exposition as shorthand for entering the study, whatever the criteria might be in practice.) As an example of a scenario involving two viruses that each cause a 10-fold increase in the probability that an individual has ARI symptoms, possible values for the selection probabilities of equation 5 are explained in Table 2.

**TABLE 2:** Example selection probabilities of having ARI symptoms given viral states A and B, where *M* = 1 and there is therefore no selection bias

	B = 1	B = 0
A = 1	P(S∣A,B)=1	$P(S|A,\bar{B})=0.1$
A = 0	$P(S|\bar{A},B)=0.1$	$P(S|\bar{A},\bar{B})=0.01$

There would be no selection bias in this sample of symptomatic patients since *M* = 1; alternatively, having virus A raises one’s probability of having symptoms by the same multiplicative factor regardless of having virus B, and equivalently, having virus B raises one’s odds of having symptoms by the same multiplicative factor regardless of having virus A.

If each of the viruses under investigation individually has a non-zero probability of causing symptoms and the probability of having symptoms with one virus differs depending on whether one has the other virus, one possibility is that there is a sub-multiplicative effect of viruses A and B together causing symptoms relative to their individual effects (see Table 3).

**TABLE 3:** Example selection probabilities of having ARI symptoms given viral states A and B, where the *M* < 1 (i.e., bias downward under the null) because of the sub-multiplicative effect of the viruses together causing symptoms

	B = 1	B = 0
A = 1	P(S∣A,B)=1	$P(S|A,\bar{B})=0.3$
A = 0	$P(S|\bar{A},B)=0.2$	$P(S|\bar{A},\bar{B})=0.001$

In this scenario, if the population ${OR}_{X}=1$, the sample-calculated OR is biased downward (${OR}_{Y}=M*\;{OR}_{X}=0.17$) compared to the population OR.

As a final example, if having either virus A or virus B alone did not raise the probability of having symptoms by much relative to having neither, while having both viruses greatly increased the probability of having symptoms, this could be described in Table 4.

**TABLE 4:** Example selection probabilities of having ARI symptoms given viral states A and B, where the *M* > 1 (i.e., bias upward under the null) because of the supra-multiplicative effect of the viruses together causing symptoms

	B = 1	B = 0
A = 1	P(S∣A,B)=1	$P(S|A,\bar{B})=0.3$
A = 0	$P(S|\bar{A},B)=0.2$	$P(S|\bar{A},\bar{B})=0.1$

In this case, the viruses have a synergistic effect on pathogenesis, so that their joint presence has a supra-multiplicative effect on the likelihood of having symptoms relative to the effect of each individual virus. The OR is biased upward (${OR}_{Y}=1.67$) in this scenario under the null.

As these examples illustrate, the OR estimated from the clinical sample, ${OR}_{Y}$, is unaffected by selection bias only when *M* = 1, and without knowledge of the selection probabilities, the magnitude of selection bias cannot be estimated. Previous commentaries have specifically pointed to the probability of having ARI symptoms among individuals without either virus, $P(S|\bar{A},\bar{B})$ , as likely significantly lower than that of the other selection probabilities, which would induce selection bias (16, 17). Since $P(S|\bar{A},\bar{B})$ is in the numerator of *M*, all else equal, *M* will be smaller (and thus more likely to be <1) if is small. The foregoing shows more precisely the conditions under which *M* < 1.

The examples from Tables 2 to 4 also demonstrate that a related research question of trying to infer synergy in pathogenesis based on individuals’ viral states using data from symptomatic individuals is affected by the same selection bias issue. In particular, observing ${OR}_{Y}$ > 1 in a symptomatic sample could imply that the two viruses increase the probability of symptoms more than multiplicatively (*M* > 1) but could also imply that they tend to occur together more often than expected under independence in the population (${OR}_{X}$ > 1), for example, if they tend to be common in the same demographic groups.

## RELATED STUDY DESIGNS AND RESEARCH QUESTIONS

Experimental models for viral interference have shown that coinfection or sequential infection often results in decreased replication of virus B in the presence of virus A rather than a complete blockade of virus B (3, 4). Examining viral loads in coinfections has therefore been proposed as a method to evaluate virus–virus interactions. Using a sample of symptomatic individuals, Burstein et al. restricted consideration to individuals with virus B and compared the *abundance* of virus B among individuals with and without virus A, instead of comparing the presence and absence of virus B among individuals with and without virus A. This comparison involved estimating the average quantitative PCR cycle threshold (Ct) value difference between monoinfected versus coinfected samples, adjusted for confounders (17). We show below that the same form of selection bias is likely to appear in this design. While Burstein et al. considered abundance of virus B as a continuous quantity, we illustrate for simplicity by dichotomizing virus B-infected individuals as having high or low viral loads.

Consider expected proportions under the null hypothesis of no interaction in the population. Let *P*(A) be the probability of having virus A and *P*(B) be the probability of having virus B. Individuals with virus B are further separated into “high” and “low” viral load groups based on whether their viral load of virus B is high or low with respective probabilities, *P*(H) and 1 − *P*(H). Here, a high viral load corresponds to low Ct values based on a cutoff value. The population proportion frequencies are summarized in Table 5

**TABLE 5:** Population proportion frequencies of having virus A and virus B, where individuals with virus B are separated into high vs low viral load groups

	B = high	B = low	B = 0	Total
A = 1	P(A)P(B)P(H)	P(A)P(B)[1−P(H)]	P(A)[1−P(B)]	P(A)
A = 0	[1−P(A)]P(B)P(H)	[1−P(A)]P(B)[1−P(H)]	[1−P(A)][1−P(B)]	1−P(A)
**Total**	P(B)P(H)	P(B)[1−P(H)]	1−P(B)	1

The population OR for any two columns of Table 5 (or for the first two columns collapsed vs. the third, mimicking the presence/absence study design) is one under the null hypothesis. However, in a symptomatic population, the selection probabilities again enter the calculation.

Since the analysis is restricted to individuals positive for virus B, to estimate the sample OR, element-wise multiplication is applied to the matrix values in Tables 5 and 6, and the OR of the resultant matrix, excluding the B=0 column, is calculated. The sample OR would be one under the null only if:


(6) $$\left[\frac{P(S|A,B,H)}{P(S|\bar{A},B,H)}\right]/\left[\frac{P(S|A,B,\bar{H})}{P(S|\bar{A},B,\bar{H})}\right]=1$$

**TABLE 6:** Selection probabilities, representing the probability of being in the sampled population having ARI symptoms, for each category of presence/absence of virus A and abundance of virus B

	B = high	B = low	B = 0
A = 1	P(S∣A,B,H)	$P(S|A,B,\bar{H})$	$P\left(S|A,\overset{-}{B}\right)$
A = 0	$P(S|\bar{A},B,H)$	$P(S|\bar{A},B,\bar{H})$	$P(S|\bar{A},\bar{B})$

This modified study design therefore also ultimately relies on similar assumptions for the relative pathogenicity of viruses A and B and their joint probabilities of causing symptoms, but in this case, assumptions regarding the selection probabilities are made based on the presence/absence of virus A and the relative viral abundance of virus B. Although this example reduces the analysis by Burstein et al. (17) into a binary example for their semi-quantitative viral load measure, if the selection bias issue is present in the binary case, it also applies to the case of a quantitative measure for viral load. A related limitation of this design is that it relies on a dose-response effect of the amount of virus B on virus A, that is, if the interaction is so strongly inhibitory that a high viral load of B greatly reduces the probability of virus A being detected (e.g., by decreasing the duration of infection with virus A from 7 days to 1 day), the design would miss this very significant interaction.

In summary, selection bias in co-detection prevalence studies arises because viral status and viral load likely affect the probability of symptoms and therefore the likelihood of individuals being included in the study population sampled. The OR estimated from the sampled study population could be corrected to estimate the unbiased population-level OR if the selection probabilities (i.e., the probability of having ARI symptoms for different combinations of viruses A and B) were known, but they typically are not. This premise is important because selection bias is not an issue only in the case where the cross-product of the selection probabilities equals one. Attempts to address this issue by using a semi-quantitative viral load measure for one virus are prone to the same selection bias issue. Likewise, the use of symptomatic samples to assess whether viral infections are synergistic in causing symptoms falls prey to the same bias, in the other direction: an excess of dually infected individuals among symptomatic persons compared to single infection prevalence could be due to synergy in causing symptoms, or due to potentiation of infection by one virus when one is already infected with the other, or a combination of these two.

In the next section, we propose study designs and provide sample size estimates for studies not conditioned on symptom status that may be used to ascertain virus–virus interactions.

## STUDY DESIGNS TO MEASURE VIRUS–VIRUS INTERACTIONS

Human challenge studies have been used for hundreds of years to garner greater scientific understanding of viral life cycles and pathogenesis. In human challenge studies, volunteers are intentionally infected with a pathogen at a safe infectious dose and isolated in a quarantine unit during their infection, where they receive close and continuous medical monitoring (25). To our knowledge, human challenge studies have yet to be conducted to study virus–virus interactions. It is plausible that a study could be designed such that volunteers are randomized to be inoculated with either one virus or mock inoculation, and in a few days, all volunteers are inoculated with a different challenge virus. Detailed information on symptoms progression, viral kinetics, and immune correlates of protection could be collected. This design would be analogous to the experimental studies that have investigated virus–virus interactions using organoid models (3–6, 8, 9) and animal models (7, 26), and it could help elucidate understanding of direct, within-host mechanisms of interaction, but not indirect mechanisms. Human challenge studies, however, are also associated with significant ethical considerations and require weighing prospective benefits to society relative to the higher risks posed to participants.

As another study design option, the effect of co-infection with respiratory viruses on a standardized attenuated challenge virus could be examined. A phase 4 immunogenicity study of a trivalent live attenuated influenza vaccine (LAIV) in the Gambia recruited clinically well children and found that children with asymptomatic respiratory viruses had upregulated mucosal interferon responses, which correlated with reduced replication of live attenuated influenza viruses post-challenge, compared to children without viral infections at baseline (27). Relatedly, a study of children with cystic fibrosis and their siblings found decreased replication of a LAIV strain in participants who tested positive for another respiratory virus before vaccination (28). Attenuated virus studies may however face concerns about whether their findings are generalizable to wild-type viruses.

Another valid approach to assess virus interactions in humans would be observational studies that sample from populations in a way that does not condition on symptoms. This would involve population-based community studies, where nasopharyngeal swab samples are collected weekly from all participants regardless of symptoms. Moreover, to minimize confounding, ideally, the participant age group and the time window of sample collection should be as narrow as possible, though these factors could also be adjusted for in the analysis, as we discuss below. To ensure specimen collection is done irrespective of symptoms, participants could collect specimen samples at home after adequate training, and the samples could be retrieved every few days by study administrators for analysis. The OR estimated from the sample population in this case may serve as an estimate of the population unimpeded by selection bias. Self-reported symptom status at the time of participants’ sample collection could also be collected every week. This information on symptoms could then be used to estimate the selection probabilities of equation 5*,* and ORs estimated from clinical samples could be adjusted using equation 4. Longitudinal community surveillance studies using similar study designs have been conducted in New York City (29), Utah (30, 31), and Australia (32) and have revealed prevalence values for low pathogenicity viruses that are higher than previously thought. Also, certain populations have been sampled in this way during COVID-19, and samples from these studies may be among the largest sample sets ever collected in this way. Pooled testing could potentially improve the cost-effectiveness of large studies.

While large community-based sampling studies may represent a methodologically straightforward approach to collecting unbiased data, they are expensive and have logistical challenges, such as the collection of specimen samples from volunteers. Conducting studies in settings where surveillance is already established and routinely performed, like in hospitals and clinical settings, therefore remains attractive, if individuals can be sampled irrespective of their symptom status.

For example, collecting samples from individuals admitted to the ER for reasons unrelated to ARI symptoms could generate an unbiased study population in this setting, if one assumes that these individuals have the same prevalence of respiratory symptoms as the overall population.

To provide a sample size estimate for co-detection studies, we use possible virus–virus interaction between three pairs of viruses as examples. Based on estimates of children <10 years old from a community-based sampling study in New York City, the average prevalence over two winter seasons from October to February 2016–2017 and 2017–2018 was ~18% for rhinovirus (RV), ~4% for adenovirus, ~10% for coronavirus, and ~2% for influenza (29). In a hypothesis testing framework for two viruses, virus A and virus B, we would test the null hypothesis $H_{0}:OR=1$ and alternative hypothesis $H_{1}:OR\neq 1$, using the notation:


(7) $$OR=\frac{p_{1}\left(1-p_{0}\right)}{p_{0}\left(1-p_{1}\right)}$$

where $p_{0}=P\left(B|\overset{-}{A}\right)$ and $p_{1}=P\left(B|A\right)$. The total sample size required, *n*, would then be calculated based on a two-sided hypothesis test for an OR estimated from a cross-sectional study (33):


(8) $$n=\frac{r+1}{r(\lambda -1{)}^{2}p_{0}^{\;\;2}}{\left(z_{1-\alpha /2}\sqrt{\left(r+1\right)p_{c}\left(1-p_{c}\right)}+z_{1-\beta }\sqrt{\lambda p_{0}\left(1-\lambda p_{0}\right)+{rp}_{0}\left(1-p_{0}\right)}\right)}^{2}$$

where

- r is the ratio of the number of individuals who have virus A to the number of individuals without virus A.
- λ is the ratio of the prevalence of the outcome (virus B) in the exposed (virus A positive) to the prevalence of the outcome in the unexposed (virus A negative); that is, $p_{1}/p_{0}$.
- $p_{c}$ is the average of the prevalence of the outcome (virus B) in the exposed and unexposed; $p_{c}=\frac{p_{0}(r\lambda +1)}{r+1}$.
- α is the type I error rate.
- β is the type II error rate.

**Fig 1:**
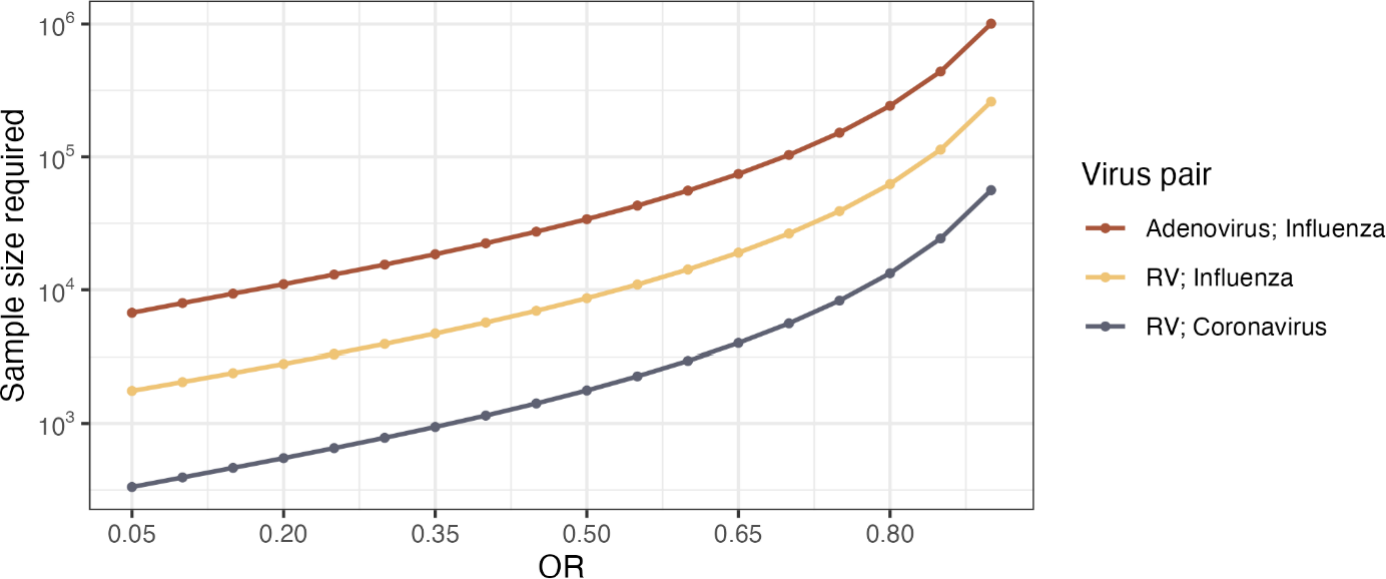
Estimated required total sample size (shown on log scale) needed to observe ORs between 0.05 and 0.9 for three example virus pairs in cross-sectional, co-detection prevalence studies.

Using an example of RV as virus A and influenza virus as virus B, we assume α=0.05; β=0.2 for 80% power; $p_{0}=$ 0.02, which is the prevalence of influenza virus in this population; and r=0.175/0.825≈0.21, based on the prevalence of RV in the population. We assume a range of OR values between 0.05 and 0.9 to reflect a potential effect size that could be detected with this study design. This range of OR values is used to calculate $p_{1}$ . The estimated required total sample size to detect viral interference between RV and influenza in children during a winter season would fall between approximately 1,760 participants for OR = 0.05 and 260,000 for OR = 0.9 (Fig. 1). For RV and coronavirus, the range is between approximately 300 and 56,000 participants based on the expected OR. The examination of two low prevalence viruses like adenovirus and influenza would meanwhile require between approximately 7,000 and 1,000,000 participants depending on the OR.

These sample size estimates are lower-bound estimates since the analysis would also need to adjust for confounding. Important confounders to consider include the age of participants and the time period over which samples are collected based on the study design and population. Additionally, if multiple study sites are used, the geographic region of the study site and specimen analysis method would also be potential confounders. These high sample size estimates indicate that studying viral interference using large population-based sampling studies may be most feasible if the magnitude of viral interference is believed to be significant and if the studies are conducted among highly susceptible populations like children during high viral circulation weeks to maximize the likelihood of capturing co-detections.

## CONCLUSION

The SARS-CoV-2 pandemic and its effect on the dynamics of other respiratory pathogens have spurred scientific interest in virus–virus interactions. Interest in virus–virus interactions also increased during the 2009 A(H1N1) pandemic, as ecological reports from several European countries suggested that high RV transmission during the summer delayed the onset of A(H1N1) outbreaks until late fall (34, 35). Various studies have been conducted using cell culture and animal models to explore direct/biological mechanisms of viral interference (3–9, 19). While these experimental studies have been instrumental in elucidating within-host mechanisms for viral interference, observing these mechanisms’ effects at the population level has been more elusive.

At the population level, studies have used time-series analyses and co-detection prevalence studies to ascertain virus–virus interactions that reflect a combination of direct and indirect, or behavior-driven, mechanisms (12, 31, 32, 36–38). Modeling studies have also been conducted to show that using cross-sectional prevalence studies to infer the impact of multivalent vaccines on type replacement may lead to biased conclusions (10, 39).

Here, we focused on selection bias issues that arise when using clinical data from symptomatic patients in co-detection studies, which is a common practice due to the expense and logistic difficulties of conducting community-wide sampling studies. A key challenge with this study design is selecting individuals irrespective of their symptom status. We provided a mechanistic interpretation of the selection bias that arises in these studies and discussed the conditions under which it would be expected. We also note that there are many other factors, mentioned briefly in Table 1 under “Confounding,” that may lead to departures from independent probabilities of infection with two viruses: any geographic and temporal differences in the circulation of two viruses will tend to cause them to infect different people, reducing coinfection, while differences in hosts such as genetics, age, and co-morbidities may make their susceptibility to different viruses positively or negatively correlated, leading to departures from independence. Multiple confounders could bias results in the same or different directions. Community-based viral sampling studies and clinical setting-based studies conducted among individuals being admitted and tested for reasons unrelated to ARI symptoms are two possibilities for unbiased study designs, and they will need to adjust for potential confounders as well. The large sample sizes estimated to be required for these studies suggest that many current efforts are underpowered to detect virus–virus interactions at the population level. Given the implications of virus–virus interactions on disease forecasting and the design and implementation of public health interventions, there is a scientific need to ascertain possible interference and its effects using unbiased designs.
